# Process optimization and production kinetics for cellulase production by *Trichoderma viride* VKF3

**DOI:** 10.1186/2193-1801-3-92

**Published:** 2014-02-17

**Authors:** Vinod Kumar Nathan, Mary Esther Rani, Gunaseeli Rathinasamy, Kannan Narayanan Dhiraviam, Sridhar Jayavel

**Affiliations:** Department of Botany and Microbiology, Lady Doak College, Madurai, 625 002 Tamil Nadu India; Centre for Environmental Studies, Lady Doak College, Madurai, 625 002 Tamil Nadu India; Department of Plant Biotechnology, School of Biotechnology, Madurai Kamaraj University, Madurai, 625 021 Tamil Nadu India; Department of Biotechnology, Directorate of Distance Education, Madurai Kamaraj University, Madurai, 625021 Tamil Nadu India

**Keywords:** Cellulase, *Trichoderma viride*, Solid state fermentation, Biomass production, Protein production kinetics

## Abstract

Microbial cellulases are the enzymes widely studied due to their enormous applications in biochemical industry. Among 12 fungal isolates isolated from mangrove plant debris and soil sample collected from Valanthakad Mangroves, Kerala, India, 3 of them were found to exhibit cellulolytic activity. Among them, the most potent isolate which exhibited maximum cellulolytic activity was identified as *Trichoderma viride* VKF3 [Gene bank accession number- JX683684.1] based on colony morphology, microscopic observation and molecular centeracterization using D1/D2 region amplification. The isolate *T. viride* VKF3 was found to be non-phytopathogenic against the selected plants. Neighbour joining tree depicted its least divergence rate from the root taxon HM466686.1. *T. viride* VKF3 was grown under dynamic carbon, nitrogen sources, pH and temperature of the medium to draw out the optimum conditions for cellulase production. Protein stability kinetics and biomass production was also studied upto 11^th^ day of incubation. It was evident from the study, that dextrose and beef extract could be used as major carbon and nitrogen sources in submerged fermentation at pH 9.0 and incubation temperature of 25°C to get maximum CMCase yield. Optimum enzyme recovery period was identified between 5^th^ to 9^th^ days of incubation beyond which the enzyme activity was reduced. By comparing two fermentation methods, submerged fermentation was found to be the best for maximum enzyme production. But utilization of substrates like sugarcane bagasse and cassava starch waste in the SSF offers a better scope in biodegradation of solid waste contributing to solid waste management.

## Background

Fungi isolated from mangrove habitats are known to be potential candidates for production of various industrially important enzymes and bio-active secondary metabolites. These enzymes are mostly ligno-cellulolytic in nature to attain their energy sources. There are many reports on the commercial applications of ligno-cellulolytic enzymes especially cellulase. *Trichoderma spp.* and *Aspergillus spp.* are two potential cellulase producers (Lynd et al. 2002). *T. viride* and *T. reesei* are two fungal strains extensively studied for their cellulase producing capability (Domigues et al. 2000, Gadgil et al. 1995).

Cellulase is a complex of three types of enzymatic complexes namely endoglucanase also called carboxymethyl cellulase, exoglucanase and β-glucosidase (Iqbal et al. 2011). It is a major enzyme used in the saccenterification of many natural substrates for production of bio-fuels. In addition to that, it is widely used for beneficial adulterations of pulp and paper centeracteristics (Kibbelwhite and Clark 1996). Other avenues for its application include cotton processing, paper recycling and as animal feed additives (Yano et al. 2012). The fungal cellulase is used for deinking of fiber surfaces in paper industries and to enhance the pulp drainage in textile industries (Penttila et al. 2004). There are many microbes capable of producing cellulase enzyme but a few of them only produces significant quantities of enzyme (Kumara et al. 2012). Apart from this, many cellulase producers are pathogenic either to plants or animals. The use of enzymes produced by these isolates might have some negative effects. Production of cellulases by the fungal isolates requires optimal conditions for their growth which leads to the release of extracellular enzymes. The growth conditions as well as extracellular enzyme production conditions is likely to vary among isolates. The major components of production medium like carbon and nitrogen sources and physical parameters like temperature, pH and incubation time were found to be critically affecting the cellulase production hence need to be optimized for every isolate (Kathiresan and Manivannan 2006, Polyanna et al. 2011). *T. viride* is a potential cellulolytic organism and widely distributed fungal species having ability to produce bio-control agents and plant growth promoting factors. The present study focuses on the optimization of various parameters for cellulase enzyme production using *T. viride* VKF3, a mangrove isolate under submerged fermentation. It would be advantageous to optimize the medium conditions for cellulase production under submerged fermentation when considering the ease in scaling up of the process for industrial applications. Meanwhile solid state fermentation offers a cost-effective production methodology for enzyme production especially using fungal systems. Diverse natural substrates are widely used with fungal strains for production of various metabolites. Most agro- wastes could be utilized as substrates for solid state fermentation. In spite of extensive study on agro-residues as solid substrates, there was no concern regarding the amount of solid waste produced after enzyme production. This also extends its application to convert solid waste into useful bio-products for commercial application thereby contributing towards solid waste management strategies.

## Results and discussion

### Screening and molecular identification of cellulolytic fungi

**Figure 1:**
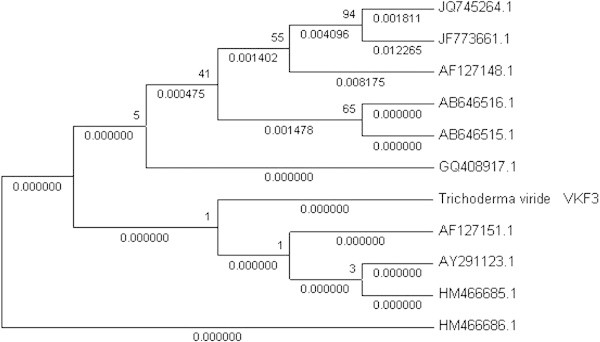
**Phylogenetic relation among** ***T. viride*** **and other fungal sequences obtained after BLASTN.**

Pure fungal colonies from mangrove soil debris were screened on CMC agar plates supplemented with congo red. Zone of clearance was observed for 3 fungal isolates among 12 isolates tested. The positive isolates were picked up and inoculated into fungal basal medium and incubated at 120 rpm at 28 ± 2°C for 3 to 5 days. Following the fifth incubation day, CMCase and FPase assay was performed for the same and VKF3 isolate was found to be a cellulolytic fungus with maximum activity. Based on the colony morphology and microscopic observation, it was identified as *Trichoderma sp.*. Further confirmation was done by molecular methods. DNA was isolated and PCR amplification of D1/D2 region was performed as suggested by Kurtzman and Robnett (1997). A 540 bp PCR amplicon was obtained after amplification and sequencing was done. Following the analysis of sequence chromatogram, BLASTN analysis was performed which confirmed VKF3 isolate as *Trichoderma viride* and the sequence was submitted in GenBank [Accession number- JX683684.1]. *T. viride* is a well known cellulolytic fungi and present isolate is from mangrove ecosystem. The cellulolytic activity of *Trichoderma sp.* was supported even by computational methods also. *T. longibrachiatum* was found to be a better cellulase producer comparing to *Clostridium thermocellum* based on its high number of active sites through computational methods (Vinod et al. 2012). Ten similar sequences were retrieved and a phylogenetic tree was constructed using neighbour joining method. The isolate was grouped among minor clade which showed the least divergence from the root taxon [Accession number- HM466686.1]. The least rate of divergence was also supported by its bootstrap value 1 (Figure 1). There are many applications reported for cellulase enzyme. Cellulase obtained from *Trichoderma viride* was used for saccenterification of waste paper materials resulting in sugar end-products (Van Wyk and Mamabolo 2013). The cellulase produced from the *T. viride* VKF3 isolate could be used in paper based industries in waste management and for deinking process also.

### Phyto-pathogenecity of fungal isolate

Phyto-pathogenecity test of the three best cellulolytic fungal isolates was done. All isolates were avirulent as there were no necrotic symptoms observed following the infection. There was no significant reduction in fresh weight and dry weight of infected plant compared to control. *T. viride VKF3* showed least negative effect compared to other two strains (Table 1). It was found from earlier studies that *T. viride* was used as a bio-control agent which also promoted the growth of certain plants. Shamalie et al. (2011) reported that *T. viride* possess growth promoting efficacy when tested on *Centella asiatica* under field trials and moreover, it had bio-control potency against nematode parasites forming root galls. Similar antagonistic potential of *Trichoderma viride* against various pathogenic fungi of *Vigna radiata* was also reported (Mishra et al. 2011). When the enzyme is produced by exploiting such avirulent microbes, it is beneficial for its safe application in saccenterification of animal fodders, in pulp modification in paper industries or even in any food processing or detergent formulations. Enzymes when used in its crude form are likely to contain fungal spores, and their release into the environment has much less negative impact when compared to enzymes derived from any pathogenic strain.

**Table 1:** **Phyto pathogenecity test for fungal isolates**

Isolates	Shoot length (cm)	Root length (cm)	Fresh weight (g)	Dry weight (g)	Necrosis at infection site
Control	12.30 ± 0.264	5.23 ± 0.152	2.43 ± 0.096	0.63 ± 0.328	No
VKF3	11.56 ± 0.709	3.83 ± 0.351	1.54 ± 0.280	0.40 ± 0.041	No

### Zymogram analysis

**Figure 2:**
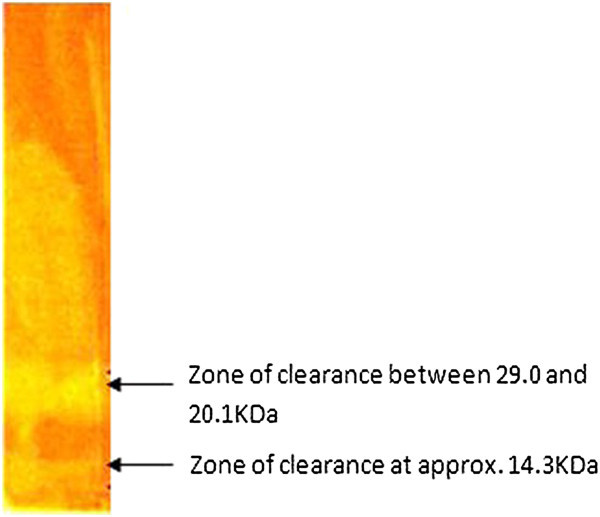
**Zymogram validating cellulolytic activity on 1% CMC supplemented gel stained with 0.1% (w/v) congo red after destaining with IM NaCl.**

Zymogram analysis using gel with CMC as substrate stained with congo red was widely used for confirmation of cellulolytic activity and to identify the fraction possessing the activity. From the analysis, two distinct zone of clearance was observed between 20.1-29.0 KDa and 14.3 KDa (Figure 2). In a similar study, three distinct bands were observed for cellulase enzyme on zymogram of thermophilic consortium with molecular masses 60, 35 and 27 KDa (Vasudeo et al. 2011).

### Carbon and nitrogen source optimization

**Figure 3:**
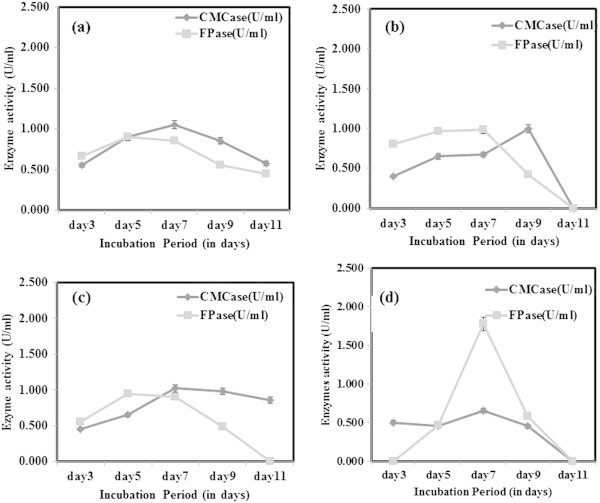
**CMCase and FPase production optimization using various carbon sources a) Dextrose b) Sucrose c) Xylose and d) CMC.**

**Figure 4:**
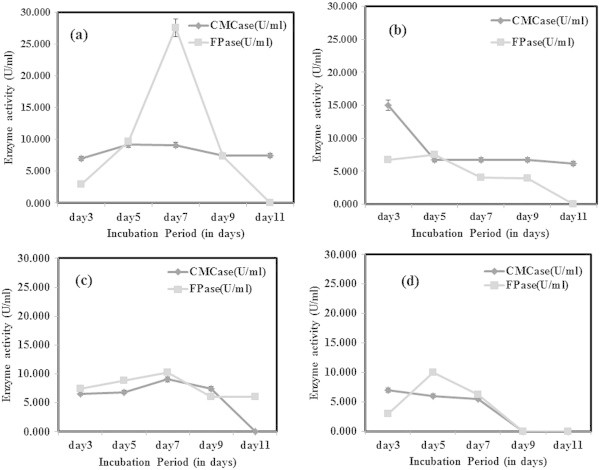
**CMCase and FPase production optimization using various nitrogen sources a) Peptone b) Beef extract c) Sodium nitrate and d) Ammonium nitrate.**

**Figure 5:**
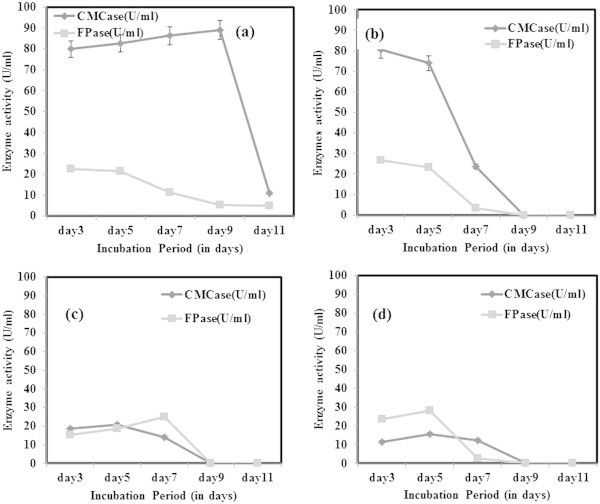
**CMCase and FPase production optimization at different incubation temperature a) 25°C b) 35°C c) 45°C and d) 55°C.**

**Figure 6:**
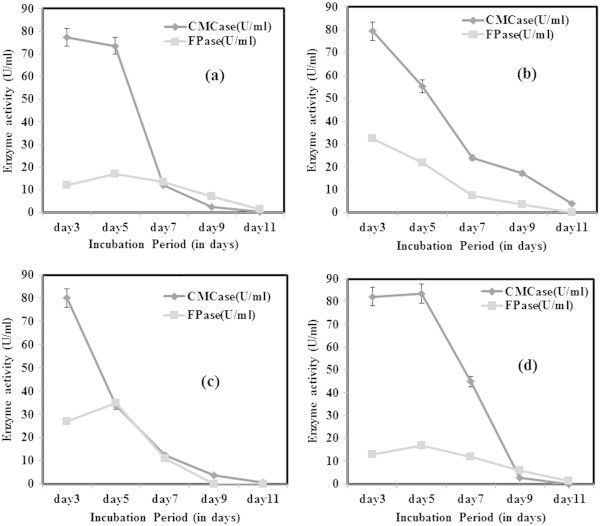
**CMCase and FPase production optimization at different medium pH a) pH 3 b) pH 5 c) pH 7 and d) pH 9.**

During optimization studies, the enzyme activity was analysed only after 3^rd^ day of incubation to allow the optimal fungal growth to be achieved. It was reported earlier that the enzyme production by the fungi started after a lag period of 24 hr or more, and the activities reached to maximal levels within 5–7 days of incubation (Gomes et al. 2006). From this study, it was concluded that for CMCase production under submerged fermentation, dextrose as the best carbon source whereas for FPase, carboxymethyl cellulose (CMC) gave better activity (Figure 3). However, CMCase production was found to be high beyond 7 days of incubation in all four carbon sources tested. Maximum FPase activity was achieved by utilizing CMC as carbon source following 7 days of incubation. But there was a decline in enzyme activity following the 7^th^ day in case of both CMCase and FPase. The decline trend of enzyme activity is likely to be due to the protease production into the medium. Ouyang et al. (2009) observed similar decline of CMCase activity from 1.01 U/ml beyond 96 hrs of incubation. However, the dextrose as best carbon source was contradicting earlier reports. Gashe (1992) achieved highest CMCase activity of 167 U/ml by *Trichoderma sp.* using CMC as carbon source. In other study on *T. reesei* C5, maximum growth and cellulase enzyme production was obtained with lactose as sole carbon source (Muthuvelayudham and Viruthagiri 2004). In nitrogen source optimization, peptone was found to be the best for FPase showing highest activity on 7^th^ day and a decline trend was observed on further incubation (Figure 4). Addition of nitrogen source like peptone was found to enhance growth and cellulase production but it was not cost effective (Chandra et al. 2009). When beef extract was used as nitrogen source, *T. viride* VKF3 produced highest CMCase activity in 3^rd^ day of incubation. But biomass production was least in beef extract in the present study which was contradicting the earlier reports. CMCase activity tends to decline after 3^rd^ day of incubation when beef extract was supplemented as nitrogen source. CMCase activity was high during incubation at 25°C on 9^th^ day whereas FPase had highest activity at 55°C on 5^th^ day of incubation (Figure 5). At neutral pH 7, CMCase exhibited highest activity on 3^rd^ to 5^th^ day of incubation followed by a rapid decline (Figure 6). FPase showed maximum activity at pH 7 on 5^th^ day of incubation. It was concluded that under submerged fermentation, a medium with CMC as carbon source and beef extract as nitrogen source at pH 7 incubated at 55°C was suitable for FPase enzyme production following 5^th^ day of incubation. Similarly medium supplemented with dextrose and peptone as carbon source and nitrogen source respectively with pH 9 incubated at 25°C achieved maximum CMCase activity beyond 5^th^ day of incubation. However, the *T. viride* VKF3 was able to produce cellulase in a better pH range from pH 3 to 9. This is beneficial for this strain to be utilized for many industrial applications. CMCase activity was high on 7^th^ day of incubation and enzyme recovery was optimal during this period beyond which there was a decline in its activity. Likewise, when medium was supplemented with CMC as carbon source and beef extract as nitrogen source with pH 7 incubated at 55°C, FPase activity was maximum on 5th day of incubation and enzyme recovery was also recommended during this period beyond which there was a decline in its activity. In a similar study, maximum FPase (0.38 U/ml) and CMCase (0.52 U/ml) were produced by *T. reesei* after 7 days of incubation. The enzyme activities decrease by further incubation. *T. reesei* produced highest level of FPase (0.48 U/ml) and CMCase (0.58 U/ml) after 9 days of incubation using wheat bran as substrate (Gomes et al. 2006).

### Protein production kinetics

**Figure 7:**
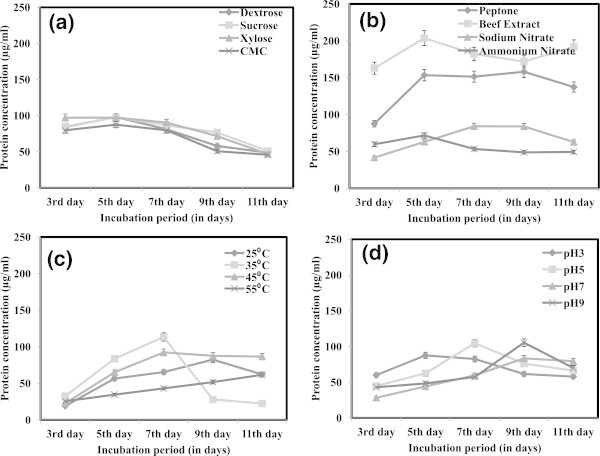
**Protein stability kinetics for various enzyme production parameters a) Carbon sources b) Nitrogen sources c) Temperature and d) pH.**

Protein production kinetics was studied by estimating protein content in the fermentation medium from 3^rd^ day to 11^th^ day of incubation by Lowry et al. (1951) method (Figure 7). In carbon source optimization, a general trend of decline in protein content was observed from 3^rd^ day onwards. Whereas in nitrogen source optimization, the protein content was quite high for medium supplemented with beef extract as nitrogen source followed by peptone. There was an increase in protein production in case of all nitrogen sources tested, and a decline was observed for incubation beyond 7^th^ day. Incubation period of 6 days was reported as optimum time period to achieve peak cellulase activity by *Trichoderma sp.* in a similar work (Sun et al. 1999). High protein production was achieved in medium incubated at 35°C following 7 days of incubation beyond which a sharp decline of protein content was observed. Though the protein production was low, there was a linear protein production kinetics observed in medium at 55°C upto 11^th^ day of incubation. Similarly, when pH was adjusted in the range of 3 and 9, protein production was higher. However, the high protein content was achieved after 7^th^ day of incubation in all pH tested.

### Biomass production

**Figure 8:**
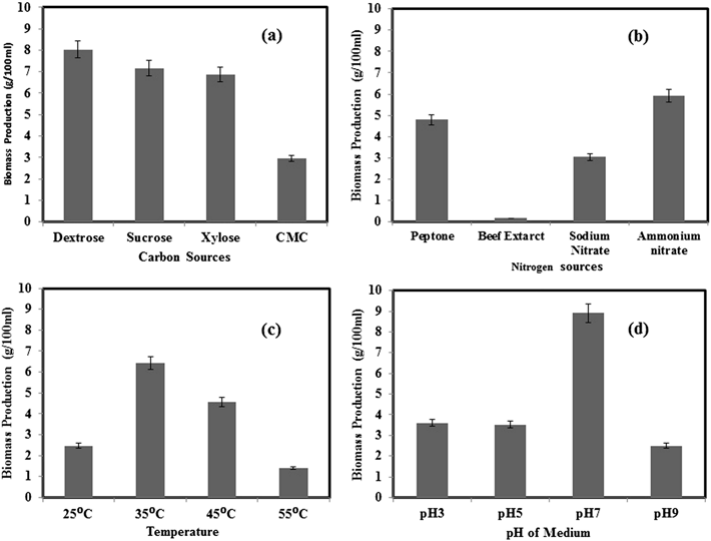
**Biomass production under various optimized conditions a) Carbon sources b) nitrogen sources c) pH and d) temperature.**

Estimation of biomass produced at each optimized parameters was done by weighing the fresh weight of fungal biomass after the 11^th^ day of incubation. High biomass of 8.025 g per 100 ml of medium was obtained when dextrose was used a carbon source (Figure 8). It was followed by sucrose, xylose and CMC. Though least biomass production was observed in case of CMC as carbon source, the FPase production was at its maximum. CMCase activity was more or less same in all other carbon sources tested. When considering the biomass production in nitrogen source optimization, ammonium nitrate showed the highest value and beef extract the least. But, the highest CMCase activity was achieved in beef extract and FPase activity in medium with peptone as nitrogen sources. This showed that there was no significant relation among biomass and enzyme production. Incubation temperature of 35°C gave maximum biomass yield whereas pH 7 gave the high biomass content in pH optimization. It was noted that FPase activity was showing an inverse relationship with the biomass production. Further, pH 7 was suitable for optimal growth as well as for enzyme production. Lower incubation temperature favoured CMCase production and high incubation temperature favoured FPase production but biomass production was highest at 35°C which was supporting its thermophilic ability.

### Solid state fermentation

**Figure 9:**
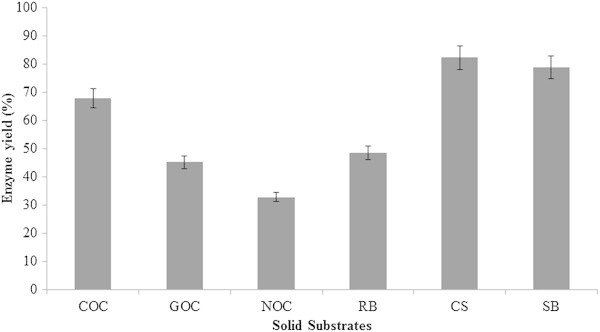
**Enzyme yield (%) of substrates used in SSF for cellulase production using** ***T. viride*** **VKF3.**

Solid state fermentation was performed using six different substrates and CMCase and FPase activity was quantified following 7 days of incubation (Table 2). Compared to the enzyme activity in the submerged fermentation, there was low activity observed in all substrates tested. However when concerning of the cost-effective production methods and its strategy of utilization of solid waste material, SSF is considered superior. In the present study, coconut oil cake was found to be a reliable substrate for CMCase production which was evident from its higher activity even at low moisture content of 20% tested. Similarly when rice bran was used as a substrate, at high moisture of 50%, it resulted in a better CMCase activity of 2.872 U/ml. Cassava starch waste yielded enzyme activity of 1.340 U/ml at 50% moisture and had maximum mass loss of 99.22%. Sugarcane bagasse when used as substrate for enzyme production using *T. viride* resulted in maximum enzyme activity of 3.229 U/ml CMCase and 1.009 U/ml FPase at higher moisture content. However the enzyme production was increasing in relation to moisture content in all the substrates tested. Gautam et al. (2011) achieved a maximum of 1.77 U/ml of exoglucanase and 1.95 U/ml of endoglucanase using *Trichoderma sp.* utilizing municipal solid waste. In another study, maximum of 0.46 and 0.26 IU/ml of CMCase and FPase respectively was obtained using *Rhizopus stolonifer* grown on cassava waste in SSF condition after 10 days of incubation (Pothiraj et al. 2006). Enzyme yield was higher for CSW followed by SB (Figure 9).

**Table 2:** **CMCase and FPase activity of enzymes produced from Solid State fermentation**

Substrates	Moisture %	CMCase activity	FPase activity	Protein concentration
		(U/ml)	(U/ml)	(μg/ml)
Coconut Oil Cake (COC)	20	2.841	0.410	204.6 ± 0.321
	30	2.883	0.218	197.3 ± 0.115
	50	3.514	0.659	284.8 ± 0.175
Groundnut Oil Cake (GOC)	20	0.294	0.110	105.4 ± 0.121
	30	0.783	0.237	213.8 ± 0.203
	50	1.009	0.455	193.7 ± 0.321
Neem Oil Cake (NOC)	20	0.625	0.101	109.3 ± 0.112
	30	1.228	0.723	189.0 ± 0.321
	50	1.289	0.812	197.0 ± 0.127
Rice Bran (RB)	20	1.136	0.562	111.2 ± 0.184
	30	1.328	0.880	171.4 ± 0.061
	50	2.872	0.982	198.7 ± 0.117
Cassava Starch (CSW)	20	1.329	0.231	233.1 ± 0.199
	30	1.340	0.881	309.2 ± 0.098
	50	1.344	0.892	314.8 ± 0.067
Sugarcane Bagasse (SB)	20	0.922	0.344	122.3 ±0.078
	30	1.382	0.873	249.0 ±0.129
	50	3.229	1.009	272.6 ±0.011

### Substrate utilization rate

**Figure 10:**
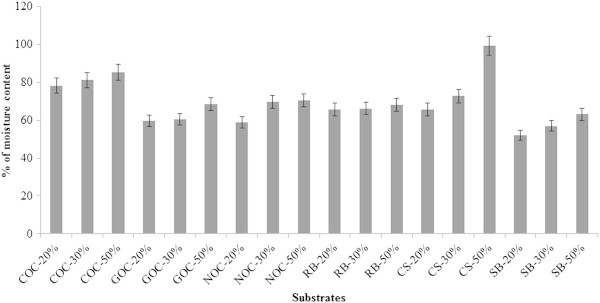
**Mass loss (%) of various substrates used in SSF for cellulase production using** ***T. viride*** **VKF3.**

When SSF is concerned, it plays an important role in solid waste management where solid substrates used are usually agro-wastes. Hence, the utilization or degradation of the solid matter is very important in this process. In spite of many researches focused on enzyme production utilizing solid waste through SSF, there was no much concern on the resultant solid residue after enzyme production. During SSF, fungal biomass utilizes solid waste for growth and further as an energy source for enzyme production. Substrate utilization was expressed in terms of mass loss (%) after the fermentation. Figure 10 shows the mass loss in various substrates after the SSF for cellulase production using *T. viride* VKF3. Highest degree of mass loss was observed in CSW followed by SB at 50% moisture content (w/v). There was an increase in mass loss percentage as the moisture was increased. In SSF, in spite of lower mass loss observed in COC when compared to SB, it gave maximal CMCase activity. In the aspect of solid waste management, agro-residues like sugarcane bagasse and coconut oil cake can be utilized for enzyme production using the present *T. viride* VKF3 isolate. However other available substrates could be used for enzyme production and the resultant solid sludge could be used for manuring as it possesses bio-control and growth promoting activity.

## Conclusion

It may be concluded from this work that under submerged fermentation, medium should be supplemented with dextrose and beef extract as carbon and nitrogen sources respectively for CMCase production. Maximum CMCase production was achieved at 25°C incubation and at pH 9. FPase production was favoured by CMC as carbon source and peptone as nitrogen source with medium pH 7 incubated at 55°C. Enzyme recovery is crucial between 5^th^ to 9^th^ days of incubation as there was a decline trend observed in general. However, *T. viride* VKF3 could be used for enzyme production and solid waste management of sugarcane and rice based industries from the evidence of better mass loss than coconut oil cake.

## Methods

### Isolation of fungal isolate

Mangrove plant debris and soil was collected from Valanthakad mangrove ecosystem, Cochin, India. The soil samples were serially diluted upto 10^-6^ dilutions and cultured on Potato Dextrose Agar (PDA) plates using spread plate method and incubated at 28 ± 2°C. Plant debris were cut into smaller pieces of length 1 cm and placed on PDA and incubated under same condition as above for 3–5 days. Distinct isolates were picked up and inoculated on the surface of fresh PDA plates for purification and further stored on PDA slants.

### Screening of cellulolytic fungi

All fungal isolates were checked for their ability to produce cellulase enzyme on PDA media supplemented with 5% carboxy methyl cellulose (CMC) (Lingappa and Lockwood 1962). Cellulolytic fungi was observed to exhibit a clear zone around the colony when medium was supplemented with 0.2% congo red and counterstained with 1 M NaCl solution after incubation for 3–5 days. Assay of positive isolates were performed to select the isolate with highest cellulolytic activity using potato dextrose broth under similar growth conditions using standard method described in the following section.

### Molecular centeracterization of fungal isolate

DNA was isolated from the fungal isolate by using the method reported earlier (Melo et al. 2006). Quality of the DNA was evaluated by spectrometric analysis as well as by performing electrophoresis on 0.8% agarose gel. DNA was further amplified using DR [5'-GGTCCGTGTTTCAAGACGG-3'] and DF [5'-ACCCGCTGAACTTAAGC-3'] universal primers for amplification of LSU 28S rDNA (Kurtzman and Robnett 1997). The PCR reaction mixture consisted of 18.7 μl deionized water, 2.5 mM Taq buffer with MgCl_2,_ 0.5 μM forward primer and reverse primers followed by 0.5 mM of dNTPs. Finally 1.2 U of Taq polymerase and 2 μl of 2 ng/ml template DNA was added and made upto 25 μl. Initial denaturation was performed at 95°C for 5 min followed by denaturation at 94°C for 30 sec. Annealing and extension was done at 55°C and 72°C respectively for 30 sec. Final elongation was done at 72°C for 10 min. Resultant PCR amplicon was purified and sequenced using automated DNA sequencing on ABI 3730xl DNA analyzer (Applied Biosystems, USA). The sequencing chromatogram was analyzed to extract the sequence and used for BLASTN analysis against non-redundant NCBI database which resulted in the identification of ten similar sequences. Clustal W multiple sequence alignment (Hompson et al. 1994) was performed using BioEdit 5.0 and phylogenetic tree was constructed for the aligned sequences in MEGA 5.0 (Tamura et al. 2011) based on neighbour joining method (Saitou and Nei 1987).

### Phyto-pathogenecity of fungal isolates

*Vigna radiata* (L.) R. Wilczek was grown in sterile soil upto 4–5 leaves stage. Incisions were made using sterile scalpel and spore suspension (10^8^ spores/ ml) was inoculated. The inoculated plantlets were incubated under moist condition for two days to develop fungal infection. Control plants were treated with sterile distilled water at incision site (Aneja 2003). After a week’s incubation under controlled conditions shoot length, root length, fresh weight and dry weight of test plants were compared with the control plant. Appearance of any visible necrosis development at site of inoculation was also noted.

### Zymogram analysis

PAGE was performed under denaturing condition. Gel electrophoresis was done on 1% (w/v) CMC and stained with 0.1% (w/v) congo red solution at room temperature for 30 min. The cellulase activity band was observed as a clear colourless area depleted of CMC, against a red background when destained in 1 M NaCl solution (Holt and Hartman 1994).

### Production medium preparation

Optimizing of conditions was performed to attain the maximum cellulase enzyme activity with the fungal isolate *T. viride* VKF3. Culture inoculum was prepared in fungal production medium with the pH of 6.5 (Jayant et al. 2011). The sterilized production medium was inoculated with *T. viride* VKF3 (10^8^ spores/ml) and incubated on a rotatory shaker at 150 rpm at room temperature for 3 days.

### Optimization of fermentation conditions

Fungal basal medium [2.0 g KH_2_PO_4_, 0.3 g urea, 0.3 g MgSO_4_.7 H_2_O, 0.3 g CaCl_2_, 5 mg FeSO_4_. 7 H_2_O, 1.6 mg MnSO_4_.H_2_O, 1.4 mg ZnSO_4_.7 H_2_O and 1.5 mg CoCl_2_.6 H_2_O in 1000 ml distilled water] was supplemented with four different carbon sources namely dextrose, sucrose, xylose and CMC. Peptone was also included as a nitrogen source and fungal inoculum of 3% was added into the sterilized medium and incubated on a rotatory shaker at 150 rpm at room temperature for 3 days. Cellulase activity was quantified from 3^rd^ day to 11^th^ day to understand the enzyme production kinetics. Similar protocol was followed for nitrogen source optimization with any one of the following nitrogen sources like peptone, beef extract, sodium nitrate or ammonium nitrate supplemented into basal medium. Cellulolytic activity was quantified by the method described in the following section. The identified best carbon source was further used for nitrogen source optimization. Four different pH and incubation temperature were tested and evaluated for optimum cellulase production. Medium pH was fixed at 3, 5, 7, 9 and incubated at temperatures 25°C, 35°C, 45°C and 55°C (best pH with best temperature). Likewise, the medium was inoculated with 3% fungal inocula and kept on a rotatory shaker. Cellulase activity was analysed from 3^rd^ to 11^th^ day of incubation.

### Solid state fermentation

Cellulase production was evaluated on abundant solid substrates to reduce the production cost. Solid state fermentation was performed using natural substrates like cassava starch waste (CSW), rice bran (RB), coconut oil cake (COC), groundnut oil cake (GOC), neem oil cake (NOC) and sugarcane bagasse (SB). The above substrates were moistened with basal fungal medium excluding the carbon and nitrogen sources. Substrates were added with 20, 30 and 50% (w/v) of basal medium and inoculated with 3% fungal inoculum. The solid state cultures were incubated at 28 ± 2°C for 5–7 days. Differences in growth rate on solid substrates were visually evaluated. Enzyme extraction was done by addition of phosphate buffer [pH 6.8] to the solid substrate followed by incubation for 3 hours. The medium was filtered through sterile mesh to obtain crude enzyme. Following the enzyme extraction, weight of the oven dried (105°C for overnight) substrates was noted and substrate utilization was expressed in terms of mass loss in percentage (Bucher et al. 2004). All the experiments were performed in triplicates unless otherwise mentioned and results were expressed as mean value ± SE. Enzyme yields were calculated using the following formula: Enzyme yield = (total amount of enzyme/enzyme activity) × 100.

### Enzyme extraction

After 7^th^ day, all solid state fermentation flasks were flooded with equal amount of sodium phosphate buffer [pH 6.8] and incubated for 1–3 hours. The solid substrate was filtered through a sterilized sieve to extract the crude enzyme solution.

### Cellulase assay

CMCase (carboxy methyl cellulase) [EC. 3.2.1.4] activity was assayed using dinitrosalicylic acid (DNS) method (Mandels and Weber 1969). Supernatant was collected by centrifuging medium at 10,000 rpm for 10 min at 4°C. 1% of CMC in 0.1 M phosphate buffer (pH 6.8) was used as the substrate. The reaction mixture had 1 ml of substrate solution and 1 ml of enzyme. The reaction was carried out at 55°C for 15 min. The amount of reducing sugar released in the hydrolysis was measured and 1 unit of CMCase activity was expressed as 1 μ mol of glucose liberated per ml of enzyme per minute.

### Filter paper-ase (FPase) assay

50 mg of dry Whatmann No. 1 filter paper discs were incubated with 0.5 ml culture filtrate obtained after centrifugation similar to the above conditions. 0.5 ml sodium citrate buffer was added and incubated for 1 hr at 50°C. The glucose liberated from the above reaction was measured by DNS method. One unit of enzyme activity was defined as the amount of enzyme required to release 1 μ mol reducing sugars per ml under standard assay condition (Gilna and Khaleel 2011).

### Protein estimation

After the incubation, the medium was centrifuged at 10,000 rpm for 10 min at 4°C and the supernatant was collected. Protein content of the supernatant was estimated as shown by Lowry et al. (1951) with BSA as the standard. 0.5 ml of the crude enzyme sample was added with equal amount of distilled water followed by addition of 5 ml of colour forming reagent and incubated at room temperature for 10 min. 0.5 ml of Folin’s reagent was added and incubated for 20 min at room temperature and absorbance was taken at 660 nm. Absorbance was compared with standard graph obtained using BSA.

### Biomass production

Biomass produced under optimum growth conditions were estimated by weighing the fresh weight of the filtered fungal biomass through sterilized sieves. Fresh weight of the biomass produced was expressed in gram per 100 ml of production medium.

## Abbreviations

PDA: Potato dextrose agar
CMC: Carboxy methyl cellulose
BSA: Bovine serum albumin
DNS: Dinitro salicyclic acid
LSU: Large sub unit
KDa: Kilo Dalton
ng: Nano gram
μl: Micro liter
ml: Milli liter
PCR: Polymerase chain reaction.
